# Clinical and demographic features associated with infections with extended-spectrum beta-lactamase–producing *Escherichia coli* in a health system in Maine, 2017

**DOI:** 10.1017/ash.2021.169

**Published:** 2021-06-28

**Authors:** Eugene W. Liu, Sarah N. Buss, Jennifer L. Trumbo, Tina M. Temples

**Affiliations:** 1 Infectious Disease, Eastern Maine Medical Center, Northern Light Health, Bangor, Maine; 2 Clinical Microbiology, Northern Light Laboratory, Northern Light Health, Bangor, Maine; 3 Clinical Research Center, Northern Light Health, Bangor, Maine

## Abstract

In this case–case control study, we identified receipt of β-lactam antibiotics and older age as independently associated with increased infection risk with ESBL-producing *Escherichia coli* among residents aged 20–88 years in a rural Maine hospital system where the infection prevalence of antibiotic-resistant *E. coli* is low.

Emergence of multidrug-resistant bacteria is thought to be associated with misuse of antibiotics. One major set of resistance genes are extended-spectrum β-lactamases (ESBLs), which produce enzymes hydrolyzing the β-lactam ring, and are commonly found in *Escherichia coli*. Prior studies have identified various risk factors for ESBL-producing bacterial infections: a history of carbapenem-resistant colonization/infection, recent international hospitalization,^1^ presence of a urinary catheter, diabetes, hospitalization in the past year,^2^ exposure to health care, previous antimicrobial use,^3^ surgery, and proton pump inhibitor (PPI) use.^4^ In Maine, the prevalence of drug-resistant *E. coli* is low (2.7% vs 7.5% nationwide).^5^ In this study, we investigated risk factors for ESBL-producing *E. coli* infections at Eastern Maine Medical Center (EMMC), a tertiary hospital, and in 4 affiliated community hospitals and outpatient clinics serving >40% of the Maine population in its rural central and northeastern regions. In this rural setting, we hypothesized that the risk of drug-resistant infection is low due to low levels of exposure to healthcare settings or antibiotics. Understanding factors contributing to a lower prevalence of ESBL-producing *E. coli* in Maine may inform efforts to prevent emergence of antibiotic-resistant bacteria.

## Methods

Study participants were identified from laboratory reports: residents of Maine aged 20–88 years with culture specimens in which *E. coli* was isolated during 2017. Individuals aged >88 years were excluded to prevent personal identification. Case patients were defined as patients with at least 1 *E. coli* isolate resistant to ceftriaxone (thus presumed to produce an ESBL). Control patients were those with *E. coli* isolates that were all susceptible to ceftriaxone.

Data elements of sex at birth, race, age, and ZIP code to determine residence in a Health Resources and Services Administration defined medically underserved area (MUA)^6^ were extracted from the Northern Light electronic medical record. For each study participant, an isolate of interest was determined, defined as the first ceftriaxone-resistant *E. coli* isolate in cases and the first ceftriaxone-susceptible isolate in controls. For each isolate of interest and corresponding specimen collection date, we extracted data on antibiotics and PPIs received in the prior 6 months (180 days), specimen type, number of primary care visits and hospitalizations of each subject in the prior 12 months, and associated diagnosis codes.

Analyses were performed using R statistical software (R Foundation for Statistical Computing, Vienna, Austria). We calculated Charlson comorbidity scores from diagnosis codes using the comorbidity package.^7,8^ We performed logistic regression using the survival package,^9,10^ first by single factors. Factors with significant odds ratios on simple logistic regression were used in multiple logistic regression with bidirectional stepwise variable selection by the Akaike Information Criterion to identify independent risk factors.

This study was approved by the EMMC Institutional Review Board with an exempt determination for which consent is not required, wherein the use of protected health information involves no more than a minimal risk to the privacy of individuals and could not practically be conducted without a waiver and use of protected health information.

## Results

### Demographic features associated with infection

We identified 60 case patients (6%) and 1,017 controls (94%) (Table 1). Male sex (OR, 1.96; 95% confidence interval [CI], 1.42–4.04) and older age (OR, 1.03; 95% CI, 1.02–1.05) were associated with infection with an ESBL-producing *E. coli* on simple logistic regression. Race was not associated with infection, with 97% of both case patients and controls identifying as white. Residence in a MUA (OR, 0.56; 95% CI, 0.33–0.95) was negatively associated with infection.

**Table 1.** Associations Between Demographic and Clinical Features of Patients and Infection With an Extended-Spectrum β-Lactamase–Producing *E. coli* Estimated With Simple Logistic Regression Models in a Case–Control Study—Maine, 2017

Factor	% of Cases	No. (N=60)	% of Controls	No. (N=1,017)	Odds Ratio (95% Confidence Interval)
**Sex**					
Female	75	(45)	85	(869)	Reference
Male	25	(15)	15	(148)	1.96 (1.42–4.04)*
Age		(60)		(1,017)	1.03 (1.02–1.05)***^ab^
**Race**					
American Indian/Alaska Native	0	(0)	1	(9)	NA
Asian	2	(1)	1	(8)	2.13 (0.26–17.31)
Black or African American	2	(1)	0	(3)	5.68 (0.58–55.44)
Multiple	0	(0)	0	(1)	NA
Native Hawaiian or Pacific Islander	0	(0)	0	(3)	NA
Other	0	(0)	0	(4)	NA
Elected not to answer	0	(0)	0	(1)	NA
White	97	(58)	97	(988)	Reference
Residence in a medically underserved area	53	(32)	67	(682)	0.56 (0.33–0.95)*^a^
**Specimen type**					
Abdomen	0	(0)	1	(8)	NA
Blood	3	(2)	2	(18)	1.87 (0.42–8.24)
Ear	0	(0)	0	(1)	NA
Extremity	0	(0)	0	(5)	NA
Not specified	0	(0)	0	(1)	NA
Pelvis	0	(0)	0	(4)	NA
Sinus	0	(0)	0	(1)	NA
Sputum	0	(0)	0	(2)	NA
Urine	97	(58)	96	(975)	Reference
Wound	0	(0)	0	(2)	NA
Primary care visits		(60)		(1017)	1.00 (0.93–1.08)
Hospitalizations		(60)		(1017)	1.29 (1.08–1.54)**
Weighted Charlson comorbidity score		(57)		(966)	1.25 (1.15–1.37)***
Receipt of proton pump inhibitors	27	(16)	11	(110)	3.00 (1.64–5.49)***

***P < .001; **P < .01; *P < .05.

^a^Covariate included in the final multiple logistic regression model by bidirectional stepwise variable selection.

^b^Covariate with significant association with infection with an ESBL-producing *E. coli* isolate in the final multiple logistic regression model.

### Clinical predictors of infection

Most *E. coli* isolates of interest in both cases and controls were from urine (97% of cases, 96% of controls) (Table 1). No isolates from other sources were associated with infection compared to isolates from the urine. The number of hospitalizations was associated with infection (OR, 1.29; 95% CI, 1.08–1.54), but not the number of primary care visits. Weighted Charlson comorbidity score (OR, 1.25; 95% CI, 1.15–1.37) and receipt of PPIs (OR, 3.00; 95% CI, 1.64–5.49) were also associated with infection.

### Antibiotic usage associated with infection

Receipt of any β-lactam in the 6 months prior to collection of an *E. coli* isolate of interest was associated with infection (OR, 5.70; 95% CI, 3.33–9.76) (Supplementary Table 2 online). Within this class of antibiotics, multiple β-lactam antibiotics were associated with infection: piperacillin/tazobactam, cefazolin, ceftriaxone, and cefpodoxime. Receipt of azithromycin (OR, 4.77; 95% CI, 1.53–14.85) was also associated with infection.

**Table 2.** Risk Factors for Infection With an ESBL Isolate, as Estimated With a Multiple Logistic Regression Model in a Case–Control Study—Maine, 2017 with 60 Cases and 1,017 Controls

Factor	Odds Ratio (95% Confidence Interval)
Age	1.02 (1.01–1.04)**
Residence in a medically underserved area	0.60 (0.34–1.04)
Receipt of β-lactam antibiotic	4.63 (2.57–8.32)***

***P < .001, **P < .01.

### Independent risk factors for infection

To identify independent risk factors, we performed multiple logistic regression with bidirectional stepwise variable selection on covariates associated with infection on simple logistic regression: sex, age, MUA residence, number of hospitalizations, weighted Charlson comorbidity score, receipt of PPIs, β-lactam antibiotics, and azithromycin. Bidirectional stepwise regression indicated that age, MUA residence, and receipt of β-lactam antibiotics should be included in our multiple logistic model. In this model, age (OR, 1.02; 95% CI, 1.01–1.04) and receipt of a β-lactam antibiotics (OR, 4.63; 95% CI, 2.57–8.32) were associated with infection, whereas MUA residence (OR, 0.60; 95% CI, 0.34–1.04) was not (Table 2).

## Discussion

In this study, we identified receipt of β-lactam antibiotics and age as independent risk factors for infection with an ESBL-producing *E. coli* isolate in a hospital system in rural Maine in 2017. This association of receipt of β-lactams and infection is consistent with the selective pressure that β-lactams apply on *E. coli* variants, allowing ESBL-producing *E. coli* to predominate and cause infection. The association with age may be a marker for past exposure to β-lactam antibiotics or nosocomial infection with ESBL-producing *E. coli* as we only examined antibiotic exposures 6 months prior to the collection date of the isolate of interest. Recent receipt of β-lactam antibiotics (within 6 months) may have selected out archived ESBL-producing *E. coli* variants, the numbers of which may be correlated with age.

Receipt of azithromycin, a non–β-lactam, was associated with infection on simple logistic regression, but not independently, and may be due to the practice of using azithromycin for empiric antibiotic coverage with β-lactam antibiotics. Similarly, the association seen with receipt of PPIs only on simple logistic regression may be due to their use as prophylaxis against stress ulcers during hospitalizations.

With respect to the degree of exposure to healthcare, although we noted an association between infection and the number of hospitalizations and Charlson comorbidity scores, and a negative association with MUA residence on simple logistic regression, none of these factors were independently associated with infection. This finding suggests confounding with receipt of β-lactam antibiotics.

This study has several limitations. We examined receipt of antibiotics over 6 months; thus, the effect of remote receipt of antibiotics is unknown. The degree to which age interacts with receipt of β-lactams also remains unclear. Furthermore, we did not account for asymptomatic carriers of ESBL-producing *E. coli* nor for treatments outside the Northern Light Health System. The mechanisms of resistance to ceftriaxone of *E. coli* isolates are unknown in this study; presumed ESBL production could arise from plasmid or chromosome mediated genes. Finally, we were unable to provide direct evidence to explain the overall decreased prevalence of drug-resistant *E. coli* in Maine.

Despite the limitations of this study, we identified receipt of β-lactams and age as independent risk factors associated with infection with ESBL-producing *E. coli*. The association of β-lactam use and ESBL-producing *E. coli* highlights the importance of antimicrobial stewardship to prevent further emergence of antibiotic-resistant bacteria.

## Supplementary material

For supplementary material accompanying this paper visit https://doi.org/10.1017/ash.2021.169. click here to view supplementary material
