# Supplementary material for: Clinical and demographic features associated with infections with extended-spectrum beta-lactamase–producing Escherichia coli in a health system in Maine, 2017
Source: Antimicrob Steward Healthc Epidemiol. 2021 Jun 28;1(1):e7. doi: 10.1017/ash.2021.169 (PMC9495424; doi:10.1017/ash.2021.169)
Supplement: Supplementary file 1 [file S2732494X21001698sup001.docx]

Supplementary Table 1. Resistance Profiles of *E. coli* Isolates of Interest in a Case–Control Study—Maine, 2017

| Antibiotic | % of Cases Resistant | No.,  n/N | % of controls resistant | No.,  n/N | χ^2^ |
| --- | --- | --- | --- | --- | --- |
| **β-lactams** |  |  |  |  |  |
| Ampicillin | 100 | 60/60 | 30 | 301/1,017 | 122.88*** |
| Ampicillin/Sulbactam | 42 | 25/60 | 13 | 132/1,016 | 35.11*** |
| Piperacillin/Tazobactam | 0 | 0/60 | 0 | 1/1,016 | 0.00 |
| Cefazolin | 100 | 60/60 | 1 | 15/1,017 | 833.75*** |
| Cefuroxime | 98 | 58/59 | 1 | 5/987 | 923.54*** |
| Cefoxitin | 16 | 9/58 | 2 | 20/986 | 32.08*** |
| Ceftriaxone | 100 | 60/60 | 0 | 0/1,017 | 1058.07*** |
| Ceftazidime | 37 | 22/60 | 0 | 0/1,016 | 362.22*** |
| Cefepime | 20 | 12/60 | 0 | 0/1,017 | 187.94*** |
| Meropenem | 0 | 0/60 | 0 | 0/1,017 | NA |
| **Fluoroquinolones** |  |  |  |  |  |
| Ciprofloxacin | 75 | 45/60 | 9 | 96/1,017 | 208.31*** |
| Levofloxacin | 73 | 44/60 | 9 | 90/1,016 | 210.14*** |
| TMP/SMX | 55 | 33/60 | 14 | 141/1,017 | 67.77*** |
| Gentamicin | 17 | 10/60 | 4 | 43/1,017 | 16.17*** |
| Nitrofurantoin | 2 | 1/58 | 0 | 4/975 | 0.18 |
| Minocycline | 5 | 3/58 | 4 | 36/974 | 0.05 |

Note. TMP/SMX, trimethoprim/sulfamethoxazole; NA, not available.

****P* < .001.

Supplementary Table 2. Associations between Receipt of Antibiotics (Both Outpatient and Inpatient) in the 6 Months (180 Days) Prior to Collection of an *E. coli* Isolate of Interest and Infection With an Extended-Spectrum β-Lactamase–Producing *E. coli* Estimated With Simple Logistic Regression Models in a Case-–Control Study—Maine, 2017

| Antibiotic | % of Cases | No. (N=60) | % of Controls | No.  (N= 1,017) | Odds Ratio  (95% CI) |
| --- | --- | --- | --- | --- | --- |
| **β-lactams** | 60 | (36) | 21 | (212) | 5.70 (3.33–9.76)***^,a,b^ |
| Ampicillin | 2 | (1) | 1 | (10) | 1.71 (0.21–13.56) |
| Ampicillin/sulbactam | 2 | (1) | 1 | (6) | 2.86 (0.34–24.11) |
| Amoxicillin | 2 | (1) | 2 | (25) | 0.67 (0.09–5.05) |
| Amoxicillin/Clavulanate | 2 | (1) | 1 | (15) | 1.13 (0.15–8.72) |
| Oxacillin | 0 | (0) | 0 | (1) | NA |
| Piperacillin/Tazobactam | 10 | (6) | 4 | (39) | 2.79 (1.13–6.87)* |
| Aztreonam | 0 | (0) | 0 | (1) | NA |
| Cephalexin | 7 | (4) | 3 | (32) | 2.20 (0.75–6.43) |
| Cefazolin | 12 | (7) | 4 | (38) | 3.40 (1.45–7.98)** |
| Cefuroxime | 3 | (2) | 1 | (13) | 2.66 (0.59–12.08) |
| Ceftriaxone | 35 | (21) | 11 | (116) | 4.18 (2.38–7.36)*** |
| Cefpodoxime | 3 | (2) | 0 | (4) | 8.73 (1.57–48.66)* |
| Cefepime | 7 | (4) | 2 | (24) | 2.96 (0.99–8.81) |
| Cefixime | 0 | (0) | 0 | (1) | NA |
| Meropenem | 2 | (1) | 0 | (4) | 4.29 (0.47–39.01) |
| **Fluoroquinolones** | 7 | (4) | 5 | (47) | 1.47 (0.51–4.24) |
| Ciprofloxacin | 2 | (1) | 2 | (25) | 0.67 (0.09–5.05) |
| Levofloxacin | 7 | (4) | 2 | (24) | 2.96 (0.99–8.81) |
| TMP/SMX | 3 | (2) | 3 | (34) | 1.00 (0.23–4.25) |
| Trimethoprim | 0 | (0) | 0 | (1) | NA |
| Gentamicin | 0 | (0) | 0 | (4) | NA |
| Nitrofurantoin | 7 | (4) | 2 | (24) | 2.96 (0.99–8.81) |
| Fosfomycin | 0 | (0) | 0 | (1) | NA |
| Vancomycin | 10 | (6) | 6 | (60) | 1.77 (0.73–4.29) |
| Linezolid | 0 | (0) | 0 | (3) | NA |
| Daptomycin | 0 | (0) | 0 | (2) | NA |
| Clindamycin | 2 | (1) | 2 | (20) | 0.84 (0.11–6.40) |
| Rifaximin | 0 | (0) | 0 | (2) | NA |
| Azithromycin | 7 | (4) | 1 | (15) | 4.77 (1.53–14.85)** |
| Metronidazole | 5 | (3) | 2 | (24) | 2.18 (0.64–7.45) |
| Bacitracin | 0 | (0) | 1 | (7) | NA |
| Neomycin | 2 | (1) | 0 | (2) | 8.60 (0.77–96.23) |

Note. CI, confidence interval; TMP/SMX, trimethoprim/sulfamethoxazole; NA, not available.

*** *P* < .001, ***P* < .01, **P* < .05.

^a^Covariate selected for inclusion in the final multiple logistic regression model by bidirectional stepwise variable selection.

^b^Covariate with significant association with infection with an ESBL producing *E. coli* isolate in the final multiple logistic regression model.
